# Effectiveness of facilitator-driven and peer-led educational interventions on tobacco use intention among adolescents: a school-based cluster randomized trial protocol

**DOI:** 10.1016/j.mex.2026.104214

**Published:** 2026-10-19

**Authors:** Sreeram RV, Rajath Rao, Nithin Kumar, Bhaskaran Unnikrishnan, Rekha Thapar, Prasanna Mithra, Ramesh Holla, Aishwarya L

**Affiliations:** a Department of Community Medicine, Kasturba Medical College Mangalore, Manipal Academy of Higher Education, Manipal, India; b Department of Community Medicine, ESIC Medical College and Hospital, Bengaluru, India

**Keywords:** Adolescents, Tobacco use, Noncommunicable diseases, Peer providers, Health education, Randomized controlled trials

## Abstract

Tobacco use remains a major preventable risk factor for non-communicable diseases (NCDs), with initiation frequently occurring during adolescence. Despite the implementation of several school-based tobacco prevention programmes, evidence on the effectiveness of peer-led educational approaches in the Indian context remains limited. This study aims to evaluate the effectiveness of a combined facilitator-driven and peer-led educational intervention in reducing the intention to use tobacco among school-going adolescents. The study will be conducted in 2 phases: Phase 1 will involve a qualitative study using in-depth interviews (IDI)with key stakeholders, including teachers, parents, psychologists, medico-social workers, students, and representatives of the national health programmes. The findings from this phase will be used to develop a culturally appropriate, evidence-based educational module. Phase 2 will comprise a school-based cluster randomized controlled trial involving 400 Class 8 students from 10 private English-medium schools in Mangalore. Schools will be randomly allocated to intervention and control arms. Both groups will receive facilitator-driven tobacco prevention education, while the intervention group will additionally receive peer-led reinforcement sessions delivered by trained peer educators. The primary outcome will be the change in intention to use tobacco, assessed using a validated Likert-scale instrument. Secondary outcomes will include changes in knowledge, attitudes, perceptions, risk perception, and patterns of tobacco use from baseline. The findings are expected to generate evidence on the effectiveness of integrating peer-led reinforcement with facilitator driven education and may inform the development of scalable and sustainable school-based tobacco prevention strategies in India.

## Graphical abstract

**Figure:**
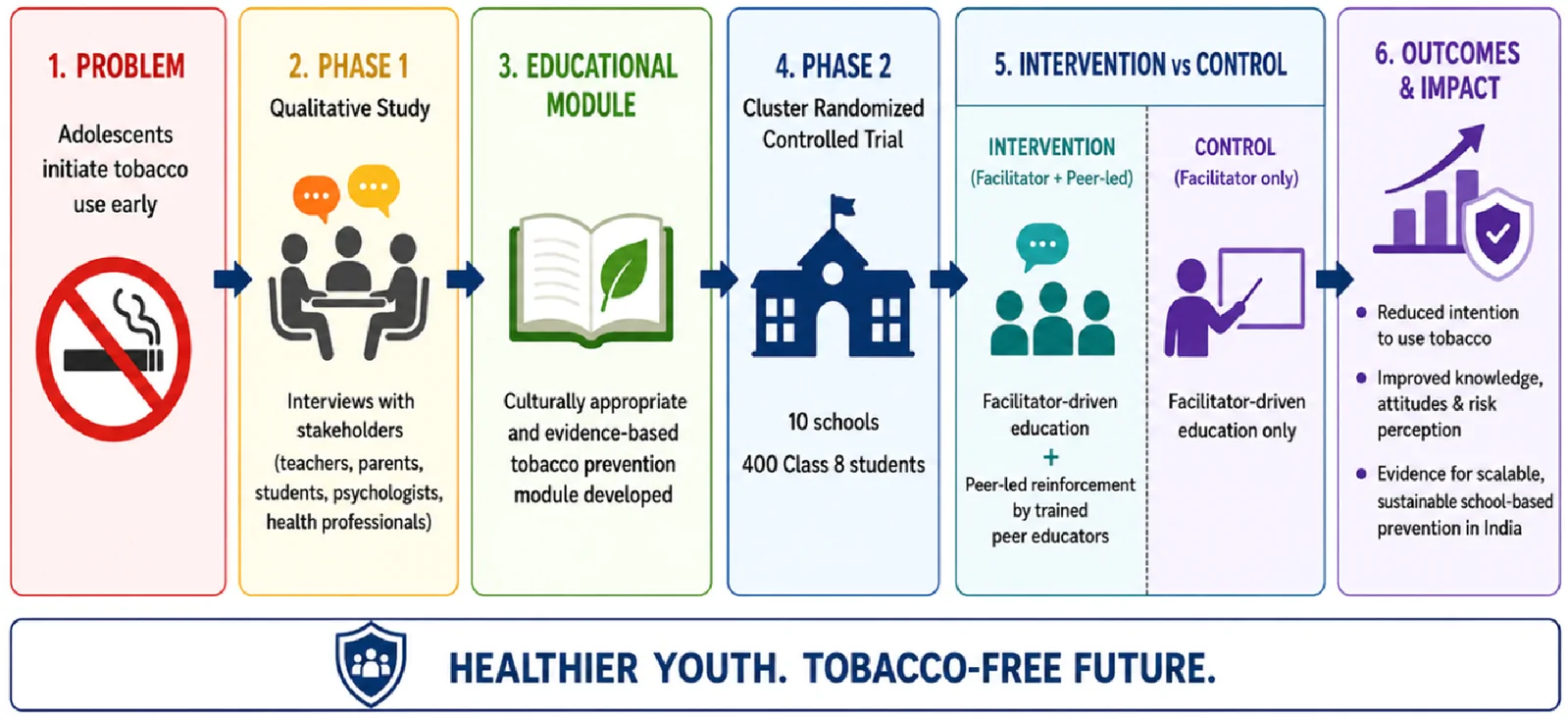

## Specifications table

**Table:** 

Subject area	Medicine and Dentistry
**More specific subject area**	*Public health*
**Name of your protocol**	Effectiveness of Facilitator-Driven and Peer-Led Educational Interventions on Tobacco Use Intention among Adolescents: A School-Based Cluster Randomized Trial Protocol.
**Reagents/tools**	*Socio-demographic details questionnaire (based on Modified Kuppuswamy classification)* *Knowledge questionnaire* *Perception and attitude scale* *Global Youth Tobacco survey questionnaire* *Risk perception scale based on Health Belief model* *Intention to use tobacco scale*
**Experimental design**	Cluster Randomized Controlled Trial
**Trial registration**	Clinical Trial Registry of India (CTRI No CTRI/2025/07/090983 dated 16 July 2025)
**Ethics**	The study protocol has been approved by the Institutional Ethics Committee (IEC) of Kasturba Medical College Mangalore, Manipal Academy of Higher Education, Manipal, India. (IECKMCMLR05/2025/269) Written informed assent will be obtained from students, along with written informed consent from the parent/guardian. Strict confidentiality will be maintained throughout the study to protect participant information and data.
**Value of the Protocol**	• ***Addresses a critical public health issue*** *by targeting adolescents before tobacco initiation, thereby contributing to the prevention of tobacco-related diseases and reducing future health and economic burdens.* • ***Generates evidence on school-based tobacco prevention strategies*** *by evaluating whether combining peer-led education with facilitator-driven sessions is more effective than facilitator-driven education alone in reducing intention to use tobacco.* • ***Provides a scalable and sustainable model for tobacco control*** *that can be integrated into school health programs and inform policymakers, educators, and public health practitioners in designing adolescent tobacco prevention initiatives.*

## Background

Non-communicable diseases (NCDs), including cardiovascular diseases, cancers, chronic respiratory diseases, and diabetes account for almost three fourths of global deaths, with majority of premature deaths occurring in low- and middle-income countries [1,2]. Among adolescents, NCDs contribute substantially to disability and disease burden [3]. In India NCDs are among the leading contributors to Disability Adjusted Life Years (DALYs) among adolescents [4]

Many behavioural risk factors for NCDs including unhealthy diet, physical activity, alcohol and tobacco are established during childhood and adolescence [5]Tobacco use is particularly concerning because of its early initiation and long-term health consequences. According to World Health Organization tobacco causes nearly nine million deaths annually worldwide, including deaths due to secondhand smoke exposure [6]. India ranks among the largest producers and consumers of tobacco with an estimated 1.3 million tobacco related death every year [7]

The increasing use of tobacco among children and adolescents is a major public health concern. The Global Youth Tobacco Survey (GYTS) India 2019 reported that 8.4% of school going adolescents aged 13–15 years use tobacco [8]. Several factors contribute to adolescent tobacco use including peer influence, curiosity, stress, parental tobacco use low risk perception, easy availability and exposure to tobacco marketing [9]

Among the various strategies to promote adolescent health, peer-led interventions have gained significant attention due to their potential effectiveness in influencing adolescent behavior. Adolescents are more likely to trust, relate to, and be influenced by their peers than by authority figures like teachers or health professionals. Schools, being structured environments offer an ideal setting for implementing health education programs. Several school-based interventional studies have demonstrated the potential of educational programmes to improve tobacco-related outcomes among adolescents. The study by Mohammadi. et.al [10]. and the MYTRI (Mobilizing Youth for Tobacco-Related Initiatives in India)[11] have reported significant improvements in tobacco-related knowledge and normative beliefs, as well as reductions in intentions to use tobacco. The interventions employed interactive and participatory strategies to engage students in tobacco prevention. Mohammadi et al. [10]. incorporated structured peer-led sessions involving role play, teamwork, and classroom activities, while MYTRI [11]. adopted a multifaceted approach combining peer-led classroom activities with school-based posters, parental involvement, and peer-led activism. These findings highlight the potential value of interactive educational strategies and peer engagement in school-based tobacco prevention.

However, further evidence is needed to establish the additional effectiveness of peer-led reinforcement when integrated with facilitator-driven education in the Indian school setting.

Despite global and national data highlighting the widespread nature of adolescent tobacco use and acknowledging it to be of public health concern, high-quality, school-based interventional studies evaluating strategies to reduce the intention to use tobacco remain limited in India. In particular, evidence on the effectiveness of peer-led educational approach complementary to facilitator-led education is insufficient. Addressing this evidence gap is essential for developing effective, contextually appropriate, and sustainable tobacco prevention interventions for school-going adolescents.

In addition to evaluating educational interventions, understanding adolescents’ beliefs and perceptions regarding tobacco-related harm is essential for developing contextually relevant and effective tobacco prevention strategies. The Health Belief Model (HBM) provides a theoretical framework for understanding health-related behaviour through six key constructs: perceived susceptibility, perceived severity, perceived benefits, perceived barriers, cues to action, and self-efficacy. In the context of tobacco prevention, these constructs reflect the adolescents’ perceptions of their susceptibility to tobacco-related harm, the seriousness of its consequences, the benefits of remaining tobacco-free, the barriers to avoiding tobacco use, the cues that motivate to avoid tobacco, and their confidence in resisting tobacco use. These constructs will guide the assessment of students’ tobacco-related beliefs and perceptions in the present study [12]

With this background, the present study aims to evaluate the effectiveness of a combined peer-led and facilitator-driven educational intervention, compared with facilitator-driven education in reducing the intention to use tobacco and improving the tobacco-related knowledge, attitudes, and practices among school-going adolescents in India. The combined intervention is based on the rationale that facilitator-led sessions can provide structured, evidence-based, and standardized information, while peer-led reinforcement may enhance the relevance, acceptability and sustained engagement of adolescents with tobacco prevention information. The study will specifically assess whether adding a peer-led component to a facilitator-led education provides an incremental benefit in reducing the intention to use tobacco.

Accordingly, both the intervention and control groups will receive facilitator-driven tobacco prevention education, while the intervention group will additionally receive peer-led reinforcement sessions delivered by trained peer educators This design will enable the evaluation of the additional benefit of peer-led reinforcement beyond that achieved through facilitator-led education alone. Although peer influence provides the theoretical rationale for incorporating peer-led sessions, it not be measured as a separate construct in the present study.

## Description of protocol

The trial protocol adheres to the Standard Protocol Items Recommendations for Interventional Trials (SPIRIT) 2025 guidelines [13] for cluster randomized trials.

**Objectives**

**Phase-1 (Qualitative study)**

To develop a comprehensive educational module for preventing tobacco use among adolescents.

**Phase-2 (Cluster Randomized Control Trial)**

**Primary Objective:**

To evaluate the effectiveness of the combined peer-led and facilitator-driven educational intervention compared to the facilitator-led educational intervention alone, in reducing the intention to use tobacco among class 8 students.

**Secondary Objectives:**

1. To determine the baseline prevalence, patterns, and correlates of tobacco use among class 8 students.
2. To assess the changes in knowledge, perceptions, and attitudes related to tobacco use among class 8 students from baseline to endline and compare these changes between the intervention and control groups.
3. To compare the risk perception regarding tobaccouse among class 8 students across the same time frame between the intervention and control groups.

**Study setting**
Mangalore, a major port city in the South Indian State of Karnataka is an important educational hub with numerous educational institutions, medical schools and engineering colleges. Schools in the city are managed by the government or private trusts and are affiliated with various educational boards including Karnataka State Board, Indian Certificate of Secondary Education (ICSE), Central Board of Secondary Education (CBSE), and the National Institute of Open Schooling (NIOS) boards [14]. The present study will be conducted in two phases in selected private English-medium schools in Mangalore City.

## Phase 1

### Study design, duration, and participants

Phase 1 will involve a qualitative study using In-depth interviews (IDI) to explore stakeholder perspectives and inform the development of the educational intervention module. Around 8–10 key stakeholders will be purposively selected to ensure representation of diverse perspectives, including parents, teachers, medico-social workers (MSWs), psychologists, student peer leaders, and programme officers associated with the National Tobacco Control Programme (NTCP). Individuals with potential conflicts of interest that could influence their perspectives on tobacco prevention, including tobacco manufacturers, distributors, vendors, and farmers engaged in tobacco cultivation will be excluded. The qualitative phase is expected to be completed over a period of six months.

### Data collection tool and methodology

In-depth interviews will be conducted using structured interview guide developed after an extensive literature review and consultation with subject experts. Two separate interview guides will be prepared in English: one for student participants and another for other key informants. The guides will be pilot tested through interviews with one student and one medico-social worker (MSW), neither of them will be included in the final study sample. Based on the pilot interviews, the guides will be modified, if necessary, to improve the clarity, sequencing and relevance of the questions.

With participants consent, interviews will be audio-recorded using a mobile phone recording device. To minimize the risk of data loss, a backup recording will be simultaneously made using co-investigator’s mobile phone. The investigator will maintain a detailed field notes and reflexive journal throughout the data collection process. Field notes will document the interview context, non-verbal cues, and other contextual observations, while the reflexive journal will record the researcher's assumptions, expectations, interactions with participants, emerging interpretations, and decisions made during data collection. For participants who decline audio recording, the co-investigator will take detailed notes as comprehensively and as close to the participants’ own words as possible, to ensure accurate representation of their perspectives. Interviews will be conducted at locations convenient to the participants, such as their workplaces or schools ensuring privacy, confidentiality, and minimal disturbance.

### Data analysis

All audio-recorded interviews will be transcribed verbatim and analysed systematically to develop codes and themes grounded in participants' narratives. Interviews conducted in Kannada language will be translated into English wherever necessary, with the translations cross-verified by a bilingual co-investigator to ensure linguistic accuracy and conceptual equivalence. Data will be analyzed using a thematic network framework, guided by a combination of deductive and inductive approaches. Deductive coding will be based on study objectives, while inductive codes will emerge from participants verbatim narratives allowing newer concepts and patterns. The analysis will involve familiarization with data, generation of initial codes, grouping of related codes into categories, development of themes, review and refinement of themes, and final interpretation of the findings [15]

Themes will be developed to capture the key perspectives of stakeholders regarding adolescent tobacco use and the development of the educational intervention module.To ensure inter-coder reliability, a subset of transcripts will be independently coded by two investigators, and discrepancies will be discussed and resolved through consensus, or consultation with a third investigator, wherever necessary. Data saturation will be considered to have been reached when no new codes relevant to study objectives emerge from subsequent interviews. Data triangulation will be achieved by interviewing diverse stakeholder groups enabling a multi-dimensional understanding of adolescent tobacco use and tobacco prevention strategies. Field notes will supplement the interpretation of interview transcripts by providing contextual information and observations recorded during data collection. For participants who decline audio recording, detailed written interview notes will serve as the primary data source, with responses documented as comprehensively and as closely to participants’ own words as possible. The investigator will maintain a reflexive journal to document assumptions, emerging interpretations, and analytical decisions throughout data collection and analysis. Themes will be reviewed against the available transcripts and detailed interview notes and supported by relevant participant quotations wherever available. Contradictory and divergent perspectives will also be considered during theme development to ensure that the findings adequately represent the range of stakeholder experiences and views.

### Expected outcome

1. Themes and subthemes related to patterns, perceptions, and determinants of tobacco use among adolescents as identified through qualitative analysis.
2. Themes and subthemes related to perceived barriers and facilitators of tobacco use prevention.
3. Stakeholder-generated recommendations for designing and implementing effective adolescent tobacco prevention interventions in schools and communities.

### Educational module

A comprehensive, multi-component educational module will be developed using a triangulated approach, that integrates findings from in-depth interviews, evidence from an extensive review of national and international literature, and recommendations from public health professionals. This approach will ensure that the module is evidence-based, contextually relevant, and aligned with established best practices in tobacco prevention and health promotion.

The module will be developed as a structured Word document to serve as the master content resource which will be subsequently adapted into a PowerPoint-based audio-visual presentation for use during the intervention sessions. Supplementary materials, including handouts, will also be prepared to reinforce learning. The module will incorporate age-appropriate and culturally relevant content organized into thematic sections. Visually appealing slides incorporating key messages, infographics, and animations, along with short, embedded video and audio clips, will be included in the presentation to reinforce learning and sustain engagement throughout the session. Each section will conclude with concise take-home messages and discussion prompts to encourage reflection and active participation.

The module will cover the following key areas: tobacco and its various products; factors influencing tobacco initiation among adolescents including peer pressure, curiosity, social influences and media exposure; short and long-term health consequences of tobacco use; tobacco control legislation and relevant legal provisions in India; and life skills for tobacco prevention including decision-making, assertiveness, effective communication, refusal skills, stress management and resistance to peer pressure. These components will aim to enhance adolescents’ knowledge, strengthen their skills to resist tobacco initiation, and promote healthy behaviours and informed decision-making.

## Phase 2

### Study design, participants and duration

Phase 2 of the study will be a cluster randomized controlled trial (Cluster RCT) conducted in 10 English-medium schools in Mangalore, with a total sample of 400 students, comprising of 200 participants in each study arm. The study population will consist of students studying in class 8 who provide written assent and whose parents or legal guardians provide written informed consent for participation.

Class 8 students will be selected as the target population as they are more likely to remain in the same school throughout the study period, facilitating follow-up and minimize attrition, compared with students from higher grades, who frequently transition to Pre-University Colleges (PUCs) after completing Class 10. Students in the class 8 are also typically in the age group of 13–14 years corresponding to early adolescence which is a crucial developmental period during which attitudes, beliefs and intentions related to tobacco use are formed, and experimentation with tobacco product may commence. At this stage, the adolescents also possess enough cognitive maturity to understand concepts such as addiction, health consequences, and peer influence, while remaining highly receptive to educational intervention and peer-led behaviour change strategies. Class 8 students were thus considered to be the most appropriate target population for the present intervention study.

Students who are absent during the baseline assessment will be excluded from the study. The trial will be conducted over 18 months, including baseline assessment, implementation of the intervention, and follow-up evaluations to assess its effectiveness.

### Sample size calculation

The sample-size assumptions were informed by the six-month post-intervention intention-to-use-tobacco scores reported by Mohammadi et al.¹⁰ (intervention: 7.73 ± 0.53; control: 6.46 ± 0.71; mean ± standard deviation). Based on these values, the standardized effect size (Cohen’s d) was calculated using the difference between the group means divided by the pooled standard deviation, yielding an estimated effect size of 2.04.

However, as this represented a very large effect, a more conservative effect size of 0.5 was adopted for sample-size estimation to avoid overestimating the expected intervention effect and to ensure adequate statistical power under more modest and realistic effect assumption. A sample size of 168 students per group was thus calculated assuming a minimum effect size of 0.5 at 90% power and 95% confidence interval and considering a design effect of 2 for cluster randomised controlled trial. After adjusting for an anticipated 10% non-response rate, the sample size was increased to 192 students per group. For operational feasibility, the final sample size was rounded upto 200 students per group, resulting in a total sample of 400 students.

### Randomization

Cluster randomization will be adapted with the school serving as the unit of randomization. An official list of all high schools in Mangalore will be procured from the Block Educational Office (BEO), from which a separate list of private English-medium high schools will be prepared and numbered sequentially. According to the Samagra Shiksha Abhiyan (SSA) [16],. an integrated scheme for school education by the Government of India, each class should consist of a minimum of 40 students. Based on this criterion, ten English-medium schools will be selected to achieve the required sample size of 200 participants in each study arm (five schools in the intervention arm and five in the control arm).

Cluster randomization will be conducted in a 1:1 ratio, with schools allocated to either the intervention or control arm using the Sealed Envelope online randomization tool. The allocation sequence will be generated by the study statistician, while the principal investigator, with assistance from school coordinators, will enroll participants and assign them to the respective study arms according to the cluster allocation. If any school allocated to a study arm declines participation, additional randomization will be conducted from the pool of remaining eligible schools to maintain the required number of clusters.

### Sampling strategy (Fig. 1)

Within each participating school (cluster), if multiple sections of Class 8 are available, one section will be selected randomly using the lottery method to minimize selection bias. All eligible students from the selected section who meet the inclusion criteria will be included in the study. If the required sample size is not achieved, an additional section will be selected from the remaining Class 8 sections in the same school using simple random sampling and all eligible students in that section will be included in the study.

### Study tool (Table 1)

A content-validated semi-structured questionnaire will be used for data collection. The questionnaire was developed based on comprehensive literature review, GYTS domains, Health Belief Model constructs, and expert consultation.

The questionnaire will have following sections:

a) Socio-demographic details questionnaire (based on Modified Kuppuswamy classification)[17]
b) Knowledge questionnaire
c) Perception and attitude scale
d) Global Youth Tobacco survey questionnaire[7]
e) Risk perception scale based on Health belief model [12]
f) Intention to use tobacco scale

Knowledge related items were adapted and modified from the previously published studies which assessed awareness of tobacco products and health effects among school going adolescents [18]. Attitude and perception related items were also adapted from previous published studies [19]. Intention to use tobacco scale was developed based on Iranian study[10] in which 10 item questionnaire was used to comprehensively address the multi-dimensional behavioral construct of tobacco use. The Global Youth Tobacco Survey (GYTS) is a standardized, school-based survey developed by the World Health Organization (WHO) and the Centers for Disease Control and Prevention (CDC) to assess tobacco use, related attitudes, exposure, and cessation behaviors among adolescents [7]

Intention to use tobacco will be operationally defined as the total score obtained on a researcher-developed, 10-item questionnaire, with each item rated on a five-point Likert scale. The total score will range from 10 to 50 and will be analyzed as a continuous variable. Higher scores will indicate a lower intention to use tobacco, whereas lower scores will indicate a greater intention to use tobacco Table 1.

**Table 1:** Tools for data collection.

Section	Domain/Focus	No. of Items	Format / Scale	Scoring	Interpretation
**A**	Socio-demographic details (Age, gender, family income, SES, family history of tobacco use)	–	Structured questionnaire	–	SES assessed using Modified Kuppuswamy scale [17]
**B**	Knowledge regarding tobacco products, effects, and legislation	12	Multiple Choice Questions and True/False	Correct = 1 Incorrect = 0 Total: 0–12	0–4: Poor 5–8: Moderate 9–12: Good
**C**	Perceptions and attitudes towards tobacco use	10	5-point Likert scale (5 = strongly agree to 1 = strongly disagree)	Total: 10–50	Higher scores = stronger negative attitude towards tobacco
**D**	Global Youth Tobacco Survey (GYTS) WHO/CDC tool [7]	57	Structured domains (Behavior, Access, Marketing, Secondhand Smoke exposure, Cessation, Education)	Domain-wise scoring	Reported as percentages and proportions
**E**	Risk perception regarding tobacco use (Based on Health Belief Model) [12]	6 subscales	5-point Likert scale (5 = strongly agree to 1 = strongly disagree)	Subscale scores: 5–25	Higher scores = stronger perception (Susceptibility, Severity, Benefits, Barriers, Cues, Self-efficacy)
**F**	Intention to use tobacco	10	5-point Likert (5 = strongly agree to 1 = strongly disagree)	Total: 10–50	Higher scores indicate lower intention to use tobacco (greater resistance to initiation), whereas lower scores indicate higher intention to use tobacco.

### Validity and pilot testing

Content validity and contextual appropriateness of the study tool was assessed by five subject experts in Community Medicine and Public health. Based on their ratings CVI-S (Scale -Content Validity Index) was calculated. CVI scale was found to be 0.88. (acceptable). The scale-level content validity index (S-CVI) summarizes experts’ ratings of the relevance of questionnaire items and helps establish whether the tool adequately represents the content it is intended to assess. A pilot test will be conducted among 40 students with similar characteristics who will not be part of the final study sample. Internal consistency reliability will be assessed using Cronbach’s alpha for Likert-scale domains such as perception, attitude, risk perception, and intention to use tobacco. Necessary modifications will be made to the study tool before the commencement of the data collection.

### Intervention (Fig. 2)

Two arm, parallel group design:

**Intervention arm:** Combined peer led and facilitator-driven educational intervention **(Intervention A)**

**Control arm:** Facilitator-driven educational intervention **(Intervention B)**

### Peer-Led educational intervention

**Selection of Peer Educators**

Peer educators will be selected through confidential nominations by classmates. Students will be asked to nominate classmates whom they trust and feel comfortable approaching for advice. Two students receiving the highest numbers of nominations who are willing to participate will be selected, withinputs from theteacher/principal regarding their suitability for the role. If a selected student declines, the next highest-nominated eligible student will be approached. Selected peer educators will undergo a one-hour facilitator-led training session covering the educational module, use of educational materials, and group facilitation skills.

In addition, reinforcement training and supervised practice sessions will be provided after observing the first peer-led session. Peer educators will also receive simplified handouts/checklists to ensure uniformity of message delivery. Thus, training will not be limited to a single one-hour exposure but will include continued support and reinforcement.

### Delivery of sessions

The first peer-led session will be conducted under the supervision of the facilitator to ensure that peer educators are familiar with the intervention content and delivery methods. Subsequently, peer educators will conduct brief sessions lasting 15–20-minutes once every 15 days during class breaks or free periods. These sessions will incorporate presentations and group discussions. The intervention schools will receive a total of 12 in-person peer-led sessions over the intervention period. The educational intervention will be supplemented by a standardized Whatsapp reinforcement message after each session, summarizing the key learning points and reinforcing the content delivered during the in-person session, resulting in a total of 12 messages over intervention period.

### Reinforcement and monitoring

Peer educators will maintain records of the session conducted, including dates, topics covered and key activities undertaken. They will share the standardized take home messages through the class specific WhatsApp groups and serve as role models by demonstrating and promoting tobacco-free behavior.

The investigators will monitor the implementation of the intervention once every month to assess adherence to the study protocol and ensure consistency and fidelity of intervention delivery across all intervention schools.

Peer-led activities and study related WhatsApp communication will be restricted to the participating class 8 students in each intervention school. Peer educators will be instructed not to disseminate intervention material to students in other schools during the follow-up period to minimize contamination between study arms.

### Facilitator-led educational intervention

Both arms will receive a facilitator-led intervention based on the developed module. The intervention will be delivered by the principal investigator during the regular school hours as a 45-minute classroom session. The session will use a PowerPoint-based audiovisual presentation incorporating age-appropriate and culturally relevant content, visually engaging slides, infographics, animations, short, embedded audio and video clips. Supplementary materials, including handoutswill also be provided to reinforce learning.

Brief discussions, question-and-answer activities, myth-versus-fact exercises, and refusal-skills practice will be incorporated to encourage active participation and sustained engagement. The same educational content and session structure will be used in both study arms.Each study arm will receive three in-person, facilitator-led sessions: First session following the baseline assessment, followed by reinforcement sessions at three and six months. At the end of six-month intervention period, all the students in both the intervention and control arm will receive a concise educational handbook summarizing the harmful effects of tobacco and strategies for tobacco prevention and refusal.

### Data collection methodology

The study protocol has been approved by the Institutional Ethics Committee (IEC) of Kasturba Medical College Mangalore, Manipal Academy of Higher Education, Manipal, India (IEC reference number: IECKMCMLR05/2025/269; approval date:19 June 2025) The study is registered with the Clinical Trial Registry of India (CTRI registration number: CTRI/2025/07/090983; registration date: 16 July 2025)

Permission to conduct the study will be obtained from the Block Educational Officer (BEO), Mangalore Taluk, and the respective school authorities. Following the receipt of the necessary approvals, the selected schools will be visited on the dates communicated in advance.(Fig. 3)

**Fig. 1:**
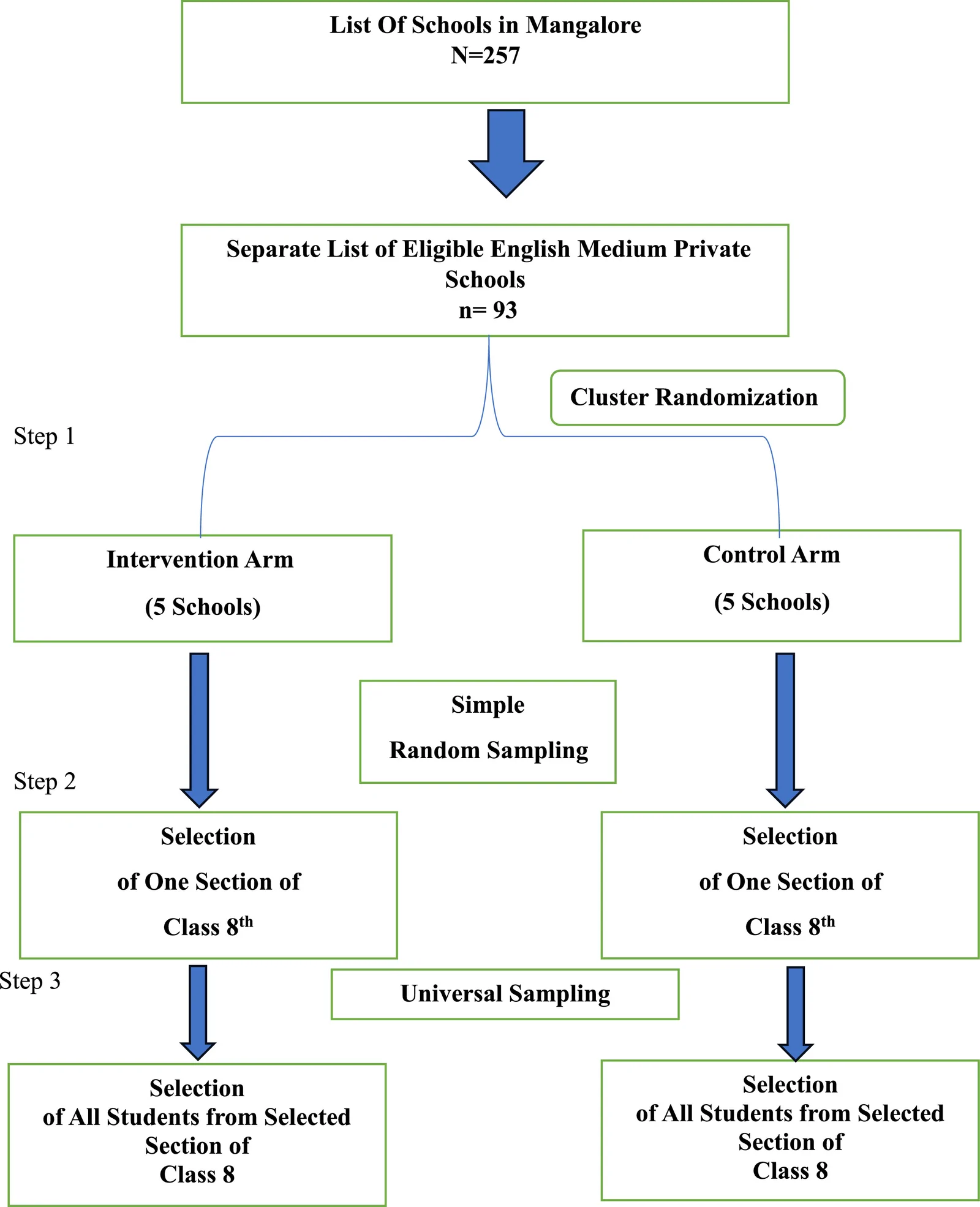
Sampling strategy.

**Fig. 2:**
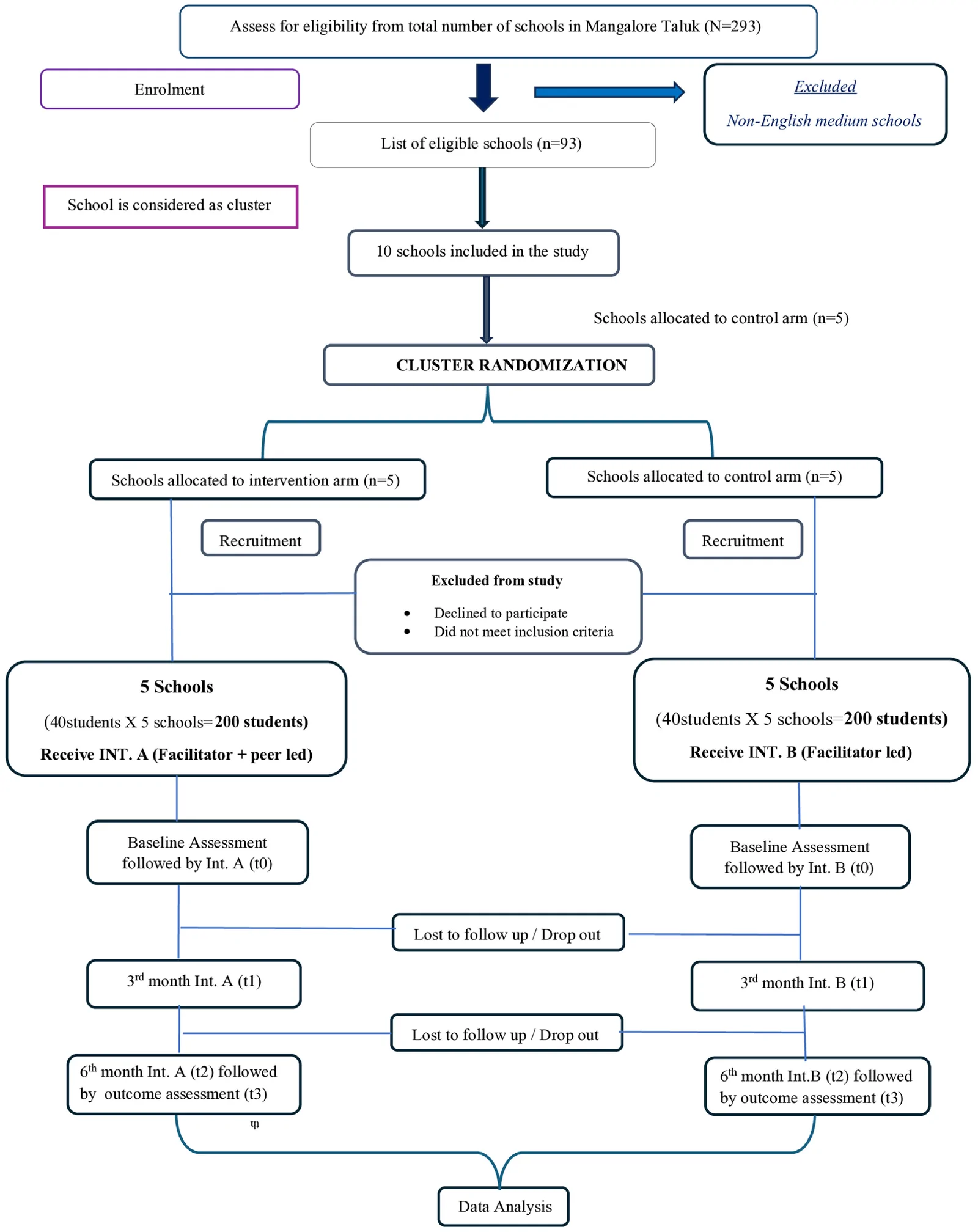
Study flow diagram.

**Fig. 3:**
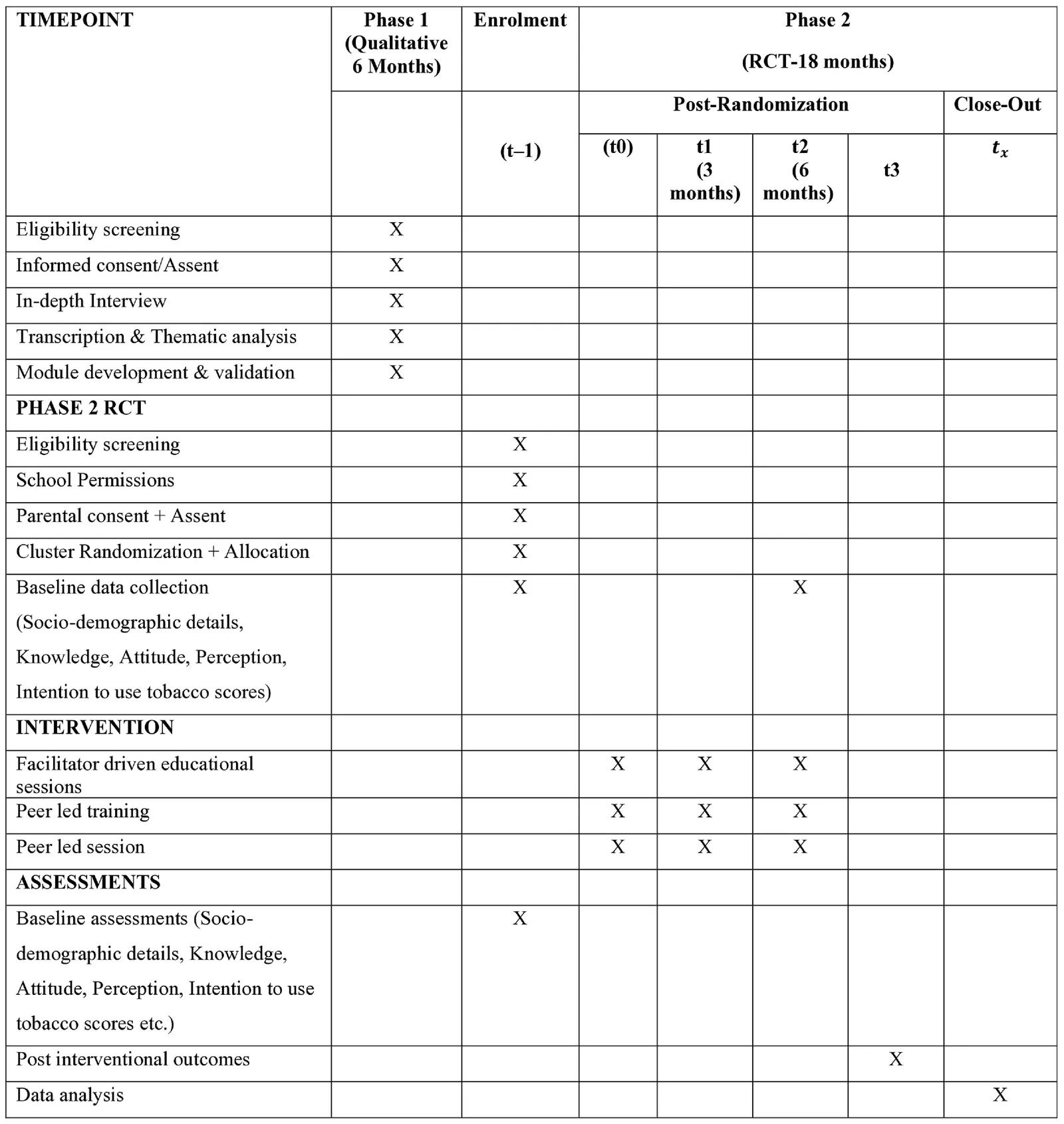
SPIRIT participant timeline: Schedule of enrolment, interventions, and assessments.13 Post test Communication.

During the first visit **(t-1)** students from class 8 at the selected schools will be briefed about the study objectives, procedures and voluntary nature of participation in English. Each student will receive a participant information sheet and parental/guardian consent form to take home. (Fig. 3) Parents or legal guardians will also receive a frequently asked questions (FAQ) sheet and will be provided with an opportunity to contact the research team for clarification. Adequate time will be allowed for returning the signed consent forms. Reminders and replacement copies will be provided through school coordinators, where necessary. Unreturned forms will not be considered as evidence of consent. Only students with documented written parental/guardian consent and their own written assent will be enrolled, and participant refusals will be respected.

During the second visit **(t0),** the principal investigator will verify the parent/guardian consent and obtain written assent from the eligible participants. Following enrollment, the participants will be provided with standard instructions on completing the baseline questionnaire which will assess the study outcomes and relevant background characteristics. (Fig.3)

Following baseline assessment, the intervention will be implemented according to the school's randomized allocation. Students in the intervention arm will receive a combination of peer-led and facilitator-led educational intervention **(Intervention A)**, whereas students in the control arm will receive only the facilitator-led educational intervention **(Intervention B) (t0).** Both groups will receive the same core educational content through the facilitator-led sessions, with the additional peer-led component provided exclusively to the intervention arm.

A reinforcement session will be conducted at three months **(t1)** and six-months **(t2)** in both the study arms. The intervention arm will receive the combined peer-led and facilitator-led intervention, while the control arm will receive the facilitator-led intervention alone. The delivery of the interventions will follow standardized content and procedures to ensure consistency across participating schools.

The primary outcome will be assessed after six-months **(t3)** in both the study arms. The effectiveness of the interventions will be evaluated by comparing the primary outcome between the two groups. Any additional tobacco-related educational activities conducted in the participating schools during the follow-up period will also be documented to identify potential co-interventions that could influence the study outcome. (Fig. 3).

### Data analysis

The data collated will be entered and analysed using IBM SPSS (Statistical Packages for Social Sciences) Statistics for Windows, Version 29.0. Armonk, NY: IBM Corp.

### Descriptive statistics

Continuous variables such as age at initiation of tobacco use, duration of use, knowledge scores, perception scores, risk perception scores, and intention-to-use scores will be summarized as mean (SD) if normally distributed, or as median (IQR) if not normally distributed. Categorical variables such as the proportion of tobacco users, pattern of use, and intention to quit will be expressed as frequencies and percentages. The results will be presented using appropriate tables and figures.

### Inferential statistics

Normality of continuous variables will be assessed using the Kolmogorov–Smirnov/Shapiro–Wilk test. For comparisons between baseline and post-intervention scores across intervention and control groups, ANCOVA (adjusting for baseline values and potential confounders) will be used for normally distributed data, while Quade’s test will be used for non-normal data. For categorical outcomes, chi-square tests or Fisher’s exact tests will be applied as appropriate.

Effect Size and Confidence Intervals

Along with p-values, effect sizes (Cohen’s d, partial eta squared, or Cramer’s V depending on the test) will be calculated with 95% confidence intervals to quantify the magnitude of intervention effects.

Handling Missing Data

An intention-to-treat (ITT) analysis will be performed to maintain the rigor of randomization. Missing data will be analyzed using complete case analysis, and sensitivity analysis may be conducted to assess the impact of missing data on outcomes.

Level of Significance

All statistical tests will be two-tailed, and results will be considered statistically significant at p < 0.05.

### Expected outcomes

The primary expected outcome is change in students’ intention-to-use tobacco scores from baseline to the six-month follow-up, compared between the intervention and control groups, to evaluate the effectiveness of the educational intervention.

The secondary expected outcome includes estimating the prevalence, assessing the patterns and correlates of tobacco use among adolescents; assessing the changes in tobacco-related knowledge, perception, attitude, and risk perception from baseline to the six-month follow up in both the study groups, and evaluating the incremental benefit of adding peer-led education to facilitator-led education in improving students’ tobacco-related knowledge, perceptions, attitudes, and intentions to use tobacco.

In the intervention group, the facilitator will conduct brief reinforcement and feedback sessions to revisit key messages covered during the educational session. Peer educators will continue to model tobacco-free behaviors and provide informal, supportive encouragement to their classmates to promote healthy choices and reinforce the intervention messages.

To minimize contamination between study arms, the peer-led intervention will not be introduced in the comparison schools during the follow-up period. On completion of the follow up period at 6 months, the peer-educational intervention will also be introduced in the comparison schools.

A summary report will also be shared with parents, teachers, and school administrators which will present the overall findings, changes observed in students’ tobacco-related knowledge, perceptions, attitudes, and intentions, and areas requiring further attention, all the while maintaining the confidentiality of the students. The report will inform future tobacco prevention and health promotion activities in schools.

### Protocol validation

Tobacco use among adolescents remains a significant public health concern in India and globally, especially due to its association with long-term noncommunicable diseases (NCDs) [7,20]. Tobacco is also known as gateway to other forms of substance abuse [9]. The early age of initiation, along with strong social influences such as peer pressure, targeted marketing by tobacco multi-national companies, and normalization of tobacco use, emphasizes the urgent need for effective and age-appropriate preventive strategies. Various studies have shown implementing the preventive strategies during early adolescence can greatly lower the risk of tobacco use [21,22]. Schools provide an ideal setting for such interventions, as they offer a structured environment and a regular access to adolescents during the formative period of their lives.

The two-phase design of this study strengthens its methodological robustness. The initial phase (Phase 1) uses a qualitative approach to develop an educational module that is contextually appropriate, culturally sensitive, and informed by multiple stakeholders. This enhances the relevance, acceptability, and feasibility of the intervention content. The implementation phase (Phase 2) employs a cluster randomized controlled trial, which is the gold standard in evaluating intervention effectiveness.

The purpose of this study is to assess the effectiveness of combined peer-led and facilitator-driven educational intervention v/s facilitator driven educational intervention on reducing the intention to use tobacco among school-going adolescents. By incorporating both facilitator-driven sessions and peer-led education, the intervention makes use of both authoritative instruction and peer influence, which are known to impact adolescent behaviour. Previous studies, including Operation MYTRI[11]. and similar international trials, [10]. have demonstrated the potential of peer-based interventions to improve knowledge, change attitudes, and decrease tobacco usage. However, there is limited high-quality, contextual evidence from Indian settings, particularly in the school environments.

## Limitations

1. **Limited generalizability:** The study is conducted among Class 8 students from private English-medium schools in urban Mangalore; therefore, the findings may not be generalizable to adolescents in government schools, rural settings, or other regions.
2. **Reliance on self-reported data:** Outcomes such as intention to use tobacco, knowledge, attitudes, and perceptions are measured through self-administered questionnaires, making the study susceptible to social desirability and reporting bias.
3. **Variability in peer educator performance:** Differences in communication skills, motivation, and engagement among peer educators may influence the effectiveness of the intervention and lead to variability in outcomes across schools.
4. **Short follow-up period:** The study assesses changes in intention to use tobacco over a limited follow-up period and may not capture the long-term sustainability of the intervention effects or actual tobacco initiation behavior.
5. **Limited number of clusters:**
. The trial includes five schools per arm because of feasibility constraints. The limited number of clusters may reduce statistical power to detect smaller between-group differences and reduce the precision of effect estimates.
6. **Contamination and external influences:**
  Cross-school interactions may lead to contamination. External educational activities, media exposure, and family or peer influences cannot be fully controlled and may affect the observed outcomes.

## Related research article

None.

## Declaration of generative AI and AI-assisted technologies in the manuscript preparation process

During the preparation of this work, the authors used ChatGPT (OpenAI) to improve the readability, grammar, language flow, and organization of the manuscript, and to assist in generating the graphical abstract. The authors reviewed and revised all AI-generated content as necessary, verified the scientific accuracy of the material, and take full responsibility for the content of the published article.

## Consent for publication

Not applicable. This study does not involve identifiable individual data.

## Dissemination

The findings of this study will be disseminated through multiple channels to ensure wide reach and practical impact. Upon completion of the study, results will be shared with participating schools and relevant educational authorities in Mangalore to inform future school-based tobacco prevention strategies. Academic dissemination will include publication in peer-reviewed journals and presentation at national and international conferences focused on public health, adolescent health, and tobacco control. A summary of key findings will also be shared with stakeholders such as teachers, parents, and local health authorities in the form of policy briefs, infographics, and community awareness materials.

The educational module, if proven effective, will be made publicly available as an open-access resource to encourage replication and scaling across other regions. The research team will also explore opportunities to integrate the intervention into existing school health. In line with open science principles, de-identified data may be shared with other researchers upon reasonable request, subject to ethical approvals and data sharing agreements.

The full trial protocol and statistical analysis plan is uploaded to The Open Science Framework (OSF). Available at https://osf.io/8e6pr/ (DOI 10.17605/OSF.IO/8E6PR)

## Data monitoring

The study will not employ a separate Data Monitoring Committee (DMC) as the interventions pose minimal risk. However, a designated research supervisor (faculty member from the Department of Community Medicine, Kasturba Medical College, Mangalore) will oversee the implementation of the study protocol, including data collection, entry, and analysis. The Principal Investigator (PI), along with the guide and co-guide only, will have full access to the final, cleaned, and locked trial dataset. This team will conduct the main analyses, prepare reports, and write the manuscript. All data will be collected using pre-tested, semi-structured questionnaires and entered into a password-protected database (Microsoft Excel and IBM SPSS v29). Periodic checks will be conducted to verify accuracy, completeness, and consistency of data. Data entry will be double-checked by two independent team members to minimize errors. Institutional Ethics Committee of Kasturba Medical College Mangalore will continuously review the research work during the study period and progress report of the research project would be would be submitted to the above committee annually.

Monitoring of intervention delivery will be performed by the primary investigator through direct observation of sessions and review of peer educator documentation (intervention logs and WhatsApp message screenshots). Any protocol deviations or adverse events, although unlikely, will be recorded and addressed promptly. Confidentiality and privacy of all participants will be strictly maintained throughout the study.

## Harms

No major risks anticipated. Any adverse events will be documented and addressed.

## CRediT author statement

**SRV:** Investigation, Methodology, Writing-Original Draft Preparation. **RR, NK:** Conceptualization, Methodology, Supervision, Writing-Original Draft Preparation, Writing-review and editing. **BUK:** Conceptualization, Methodology, Writing-review and editing. **RT, PM:** Conceptualization, Methodology, Writing-review and editing. **RH:** Methodology, Writing-review and editing. **AL:** Data analysis.

## Declaration of competing interest

The authors declare that they have no known competing financial interests or personal relationships that could have appeared to influence the work reported in this paper.

## Data Availability

No data was used for the research described in the article.
