# Supplementary material for: Effectiveness of facilitator-driven and peer-led educational interventions on tobacco use intention among adolescents: a school-based cluster randomized trial protocol
Source: MethodsX. 2026 Oct 19;17:104214. doi: 10.1016/j.mex.2026.104214 (PMC13737420; doi:10.1016/j.mex.2026.104214)
Supplement: Supplementary file 1 [file mmc1.pdf]

MANIPAL
ACADEMY of HIGHER EDUCATION
(Deemed to be University under Section 3 of the UGC Act, 1956)

Institutional Ethics Committee
Kasturba Medical College, Mangalore

(Reg. No. ECR/541/Inst/KA/2014 / RR-20)
(DHR Reg.No.EC/NEW/INST/2022/KA/0183)

Ref. No.June/2025 Date : 19.06.2025

Dear Dr. Sreeram Ravi Varma V., Dept of Community Medicine, Kasturba Medical College, Mangalore

Your research proposal was discussed in the ethics committee meeting held on 18.06.2025 and the decision is as follows:

**Protocol title:** Effectiveness of Combined Facilitator-driven and Peer-led Educational Intervention on Intention to Use Tobacco among Adolescents in Mangalore: A School based Cluster Randomized Controlled Trial
**Protocol No: IECKMCMLR05/2025/269**
**Principal Investigator**: Dr. Sreeram Ravi Varma V<br>**Guide /Co-guide/Co-investigator:** Dr. Nithin Kumar, Dr. Rajath Rao
**Name & Address of Institution:** Department of Community Medicine<br>Kasturba Medical College, Mangalore
**Review: Full review**
**Date of review:** 18/06/2025
**Decision of the Ethics Committee:**<br>> Approved
**Recommended for a period of:** study duration as mentioned in the proposal

- The approval is valid for the entire study period mentioned in the protocol.
- Your research work will be continuously reviewed by ethics committee during the study period.
- You are instructed to submit progress report of the research project annually
- Ethics committee has the right to withdraw the approval if found necessary due to protocol violations, non-compliance to regulations and guidelines
- For any modifications/changes in protocol, investigators and study site you need to submit the proposal to ethics committee and get the approval.
- You need to submit the final report and summary at the termination of the study.

Dr Sheetal Ullal
Member Secretary, IEC

KASTURBA MEDICAL COLLEGE ★ MANGALORE-575 001
INSTITUTIONAL ETHICS COMMITTEE

P.T.O

PDF Scanner ACE Scanner

Following members of the IEC were present and were involved in decision-making.

Name	Affiliations	Role in the Committee
Dr Nagesh K.R	No	Chairperson
Dr Pramod Kumar	Yes	Clinician
Dr Sheetal Ullal	Yes	Member Secretary
Dr Nutan Kamath	Yes	Clinician
Dr. Rameela Shekhar	No	Social Scientist
Dr Prasanna Mithra P	Yes	Member
Ms. Himani Kotian	Yes	Basic Scientist / Statistician
Professor. Ashalatha Pai	No	Lay Person
Mr Ranjan Rao	No	Legal Expert
Dr Sudhir Prabhu	No	Member
Dr Padma Priya J	Yes	Basic Medical Scientist
Dr Manisha N	Yes	Clinician

PDF Scanner ACE Scanner
